# Lower Cranial Nerves in the Neck: An Anatomical Study

**DOI:** 10.7759/cureus.75049

**Published:** 2024-12-03

**Authors:** João V Pinto, José Loureiro, Ricardo Vaz, Sónia Martins

**Affiliations:** 1 Otorhinolaryngology Department, Unidade Local de Saúde de São João, Porto, PRT; 2 Otorhinolaryngology Department, Faculdade de Medicina da Universidade do Porto, Porto, PRT; 3 Research Department, Centro de Investigação em Tecnologias e Serviços de Saúde (CINTESIS), Porto, PRT; 4 Anatomy Department, Faculdade de Medicina da Universidade do Porto, Porto, PRT

**Keywords:** accessory nerve, hypoglossal nerve, lower cranial nerves, neck surgery, vagus nerve

## Abstract

Objectives

The aim of this anatomical study was to analyze distances and anatomical relations between the lower cranial nerves and important neck landmarks.

Methods

Anatomical study based on neck dissection in Thiel-embalmed cadavers. Anatomical relations and distances between the vagus (X), accessory (XI), and hypoglossal (XII) nerves and important neck landmarks were registered and compared. The relation between the emergence of the great auricular nerve and the posterior border of the sternocleidomastoid muscle and certain anatomical aspects of the ansa cervicalis were also studied.

Results

A total of 18 neck dissections (seven bilateral, four unilateral) were performed on 11 adult cadavers (mean (SD) age: 74.2 (12.9) years, four male, seven female).

The X nerve was posterolateral to the common carotid artery and medial to the internal jugular vein (IJV) in 55.6% of the cases. In relation to the IJV, the XI nerve traveled mostly anteromedial (44.4%) at the level of the jugular foramen, posterior (50%) at the posterior belly of the digastric muscle, and posterolateral (77.8%) below the digastric muscle. The XII nerve was inferior (50%), medial (33.3%), and superior (16.7%) to the digastric tendon.

The distance between the XII nerve and the carotid bifurcation was significantly superior in the male gender (mean (SD): 31.7 (6.8) mm vs. 18.5 (7.9) mm, p = .003). Also, the distance between the origin of the occipital artery and the point where it crosses the XII nerve was significantly higher in females (median (IQR): 7 (12.0) vs. 4 (4.0), p = .012).

Conclusions

There is a great variability in the anatomical position, course, and distances between the lower cranial nerves and traditional anatomical landmarks in the neck. The topography of the lower cranial nerves can vary even with the gender. Proper anatomical knowledge is crucial in neck surgery to prevent potential nerve injuries.

## Introduction

Lower cranial nerve injuries are a potential complication in multiple neck surgeries such as neck dissections [1,2], mass excisions [3], cervical spine surgery [4], and carotid endarterectomy [5]. Knowledge about the anatomy of lower cranial nerves (CN) and their anatomical variations is paramount to reducing complications during these procedures.

The vagus nerve (CN X) exits the skull base through the jugular foramen and courses downward within the carotid sheath, with a variable position in relation to the internal jugular vein (IJV) and the common carotid artery (CCA) [6]. CN X injury can result in dysphonia, dysphagia, and loss of sensation in the larynx, with more symptom severity related to more proximal lesions [7].

Most frequently, the accessory nerve (CN XI) comes out of the jugular foramen, accompanying the glossopharyngeal nerve (CN IX) and CN X, and travels past the IJV laterally, with a variable relation with this vein, until it enters the deep surface of the upper portion of the sternocleidomastoid muscle (SCM). Afterward, the CN XI exits along the posterior border of the SCM and courses through the posterior neck triangle in a superficial location. Injury to this nerve can result in depression and winging of the scapula, shoulder disability, and pain [8].

The hypoglossal nerve (CN XII) exits the cranium through the hypoglossal canal and descends to reach the anterior margin of the SCM in close proximity to the internal and external carotid arteries (ICA and ECA). Subsequently, it runs transversally, superior to the hyoid bone, until it reaches the inferior surface of the tongue at the edge of the mylohyoid muscle [9,10]. Injury to this nerve may lead to dysarthria and dysphagia [10].

Anatomical variations in the course of lower neck cranial nerves are a field of surprises, and knowledge about variations and nerve distances to important anatomical landmarks may help prevent potential nerve injuries during neck surgery [11].

The aim of this anatomical study, based on cadaveric dissection, was to analyze distances and anatomical relations between important neck landmarks and the lowest cranial nerves.

This work was previously presented as a communication at the "14th meeting IJUP - Investigadores Jovens Universidade do Porto."

## Materials and methods

This was an anatomical study based on neck dissections performed in Thiel-embalmed cadavers [12]. All cadavers were provided by the Anatomy Unit of the Faculty of Medicine of the University of Porto, Portugal. Cadavers with a previous history of neck surgery or neck trauma were excluded. Some dissections were unilateral when the contralateral side had been dissected previously for another study. All dissections and measurements were performed simultaneously by two investigators. In order to simulate neck surgery, the cadaver was placed in a supine position with the head extension. A hockey-stick neck incision was used in every case, and the anatomical dissection was performed using cold instruments. All measurements were performed as early as possible in the dissection in order to keep structures close to their original position. To minimize alterations caused by extensive dissection of the neck structures, cranial nerve dissection was kept to a minimum, preserving the surrounding connective tissue and maintaining the nerves in their natural positions.

The position of the CN X relative to the IJV and the CCA was registered. The distance between the CN X and the posterior belly of the digastric muscle was measured, as described by Yigit et al. [11], from the point where the nerve becomes visible beneath the posterior belly to the posteroinferior border of the muscle. The shortest distance between the CN X and the lateral borders of the hyoid bone and the cricoid cartilage was also assessed.

**Figure 1:**
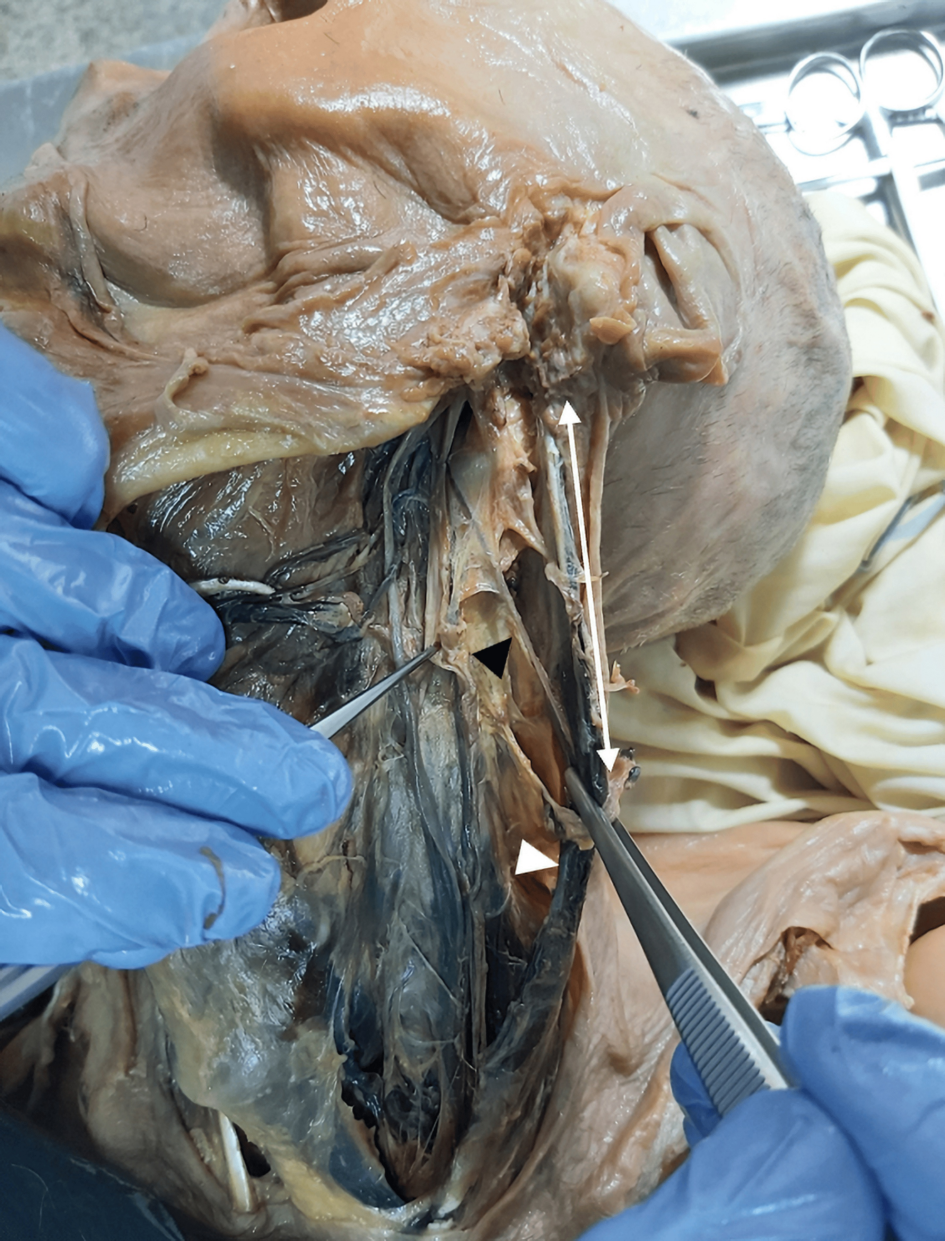
Accessory nerve dissection. black arrowhead: accessory nerve; white arrowhead: sternocleidomastoid muscle; white double-arrow: distance along the anterior border of the SCM between the mastoid tip to the point where the CN XI perforates the SCM. SCM: sternocleidomastoid muscle; CN: cranial nerve

The relative position of the CN XI with the IJV was determined at the level of the jugular foramen, the posterior belly of the digastric muscle, and inferiorly to the digastric muscle. Furthermore, the length of the CN XI from its emergence until it crosses the IJV was registered. The distance along the anterior border of the SCM between the mastoid tip and the point where the CN XI perforates the SCM was also assessed (Figure 1).

The position of the CN XII relative to the digastric muscle tendon and the shortest distances to the carotid bifurcation, the lateral border of the hyoid bone, the lateral border of the cricoid cartilage, the facial nerve (CN VII) bifurcation, the origin of the occipital artery, and the origin of the lingual artery were determined. The distances from the origin of the ansa cervicalis to the carotid bifurcation and to the angle of the mandible were also registered. Distances were measured to the nearest millimeter using a MedBAR® Skin Marker Scale (Progress Healthcare Pte Ltd., Singapore).

A descriptive analysis of the patient’s characteristics was performed, taking into consideration absolute and relative frequencies for categorical variables, mean, standard deviation, median, and range for continuous variables. The normality of continuous variables was assessed with the Kolmogorov-Smirnov test. Comparison of different variables with gender or laterality was performed with the chi-square test or Fischer’s exact test for categorical variables, the student’s T test for normally distributed continuous variables, and the Mann-Whitney U test for non-normally distributed continuous variables. Comparison of age and different variables was performed with the Kruskal-Wallis H test for categorical variables and linear regression and Pearson correlation coefficient for continuous variables. All statistical analysis was made with the IBM SPSS Statistics for Windows, Version 27 (Released 2020; IBM Corp., Armonk, New York, United States) and associations were considered significant when p<0.05.

## Results

A total of 18 dissections were performed in 11 cadavers (seven bilateral and four unilateral). Seven (38.9%) dissections were in a male neck; 11 (61.1%) in a female. The mean age was 74.2±12.9 years.

**Figure 2:**
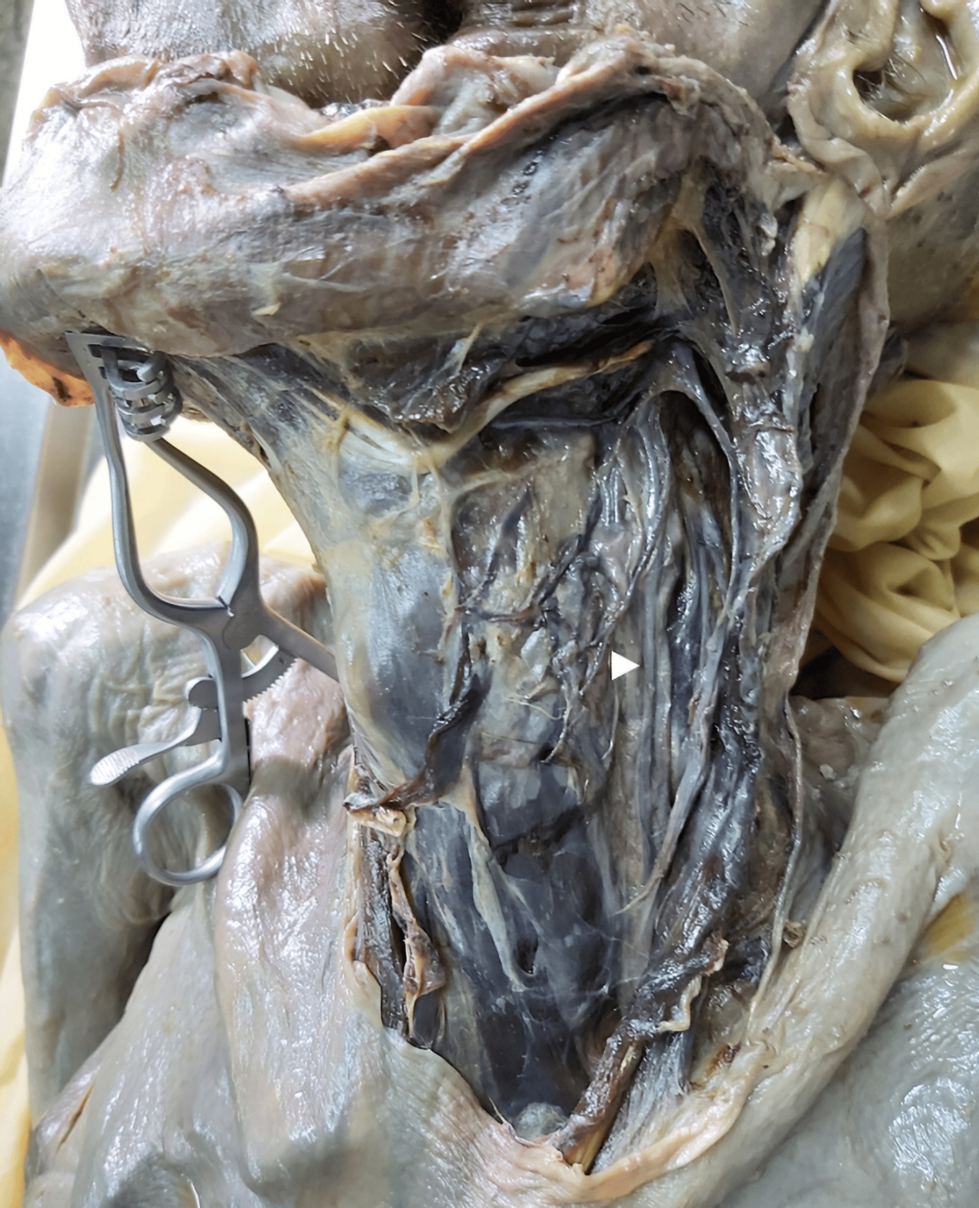
Vagus nerve dissection. white arrowhead: vagus nerve

**Figure 3:**
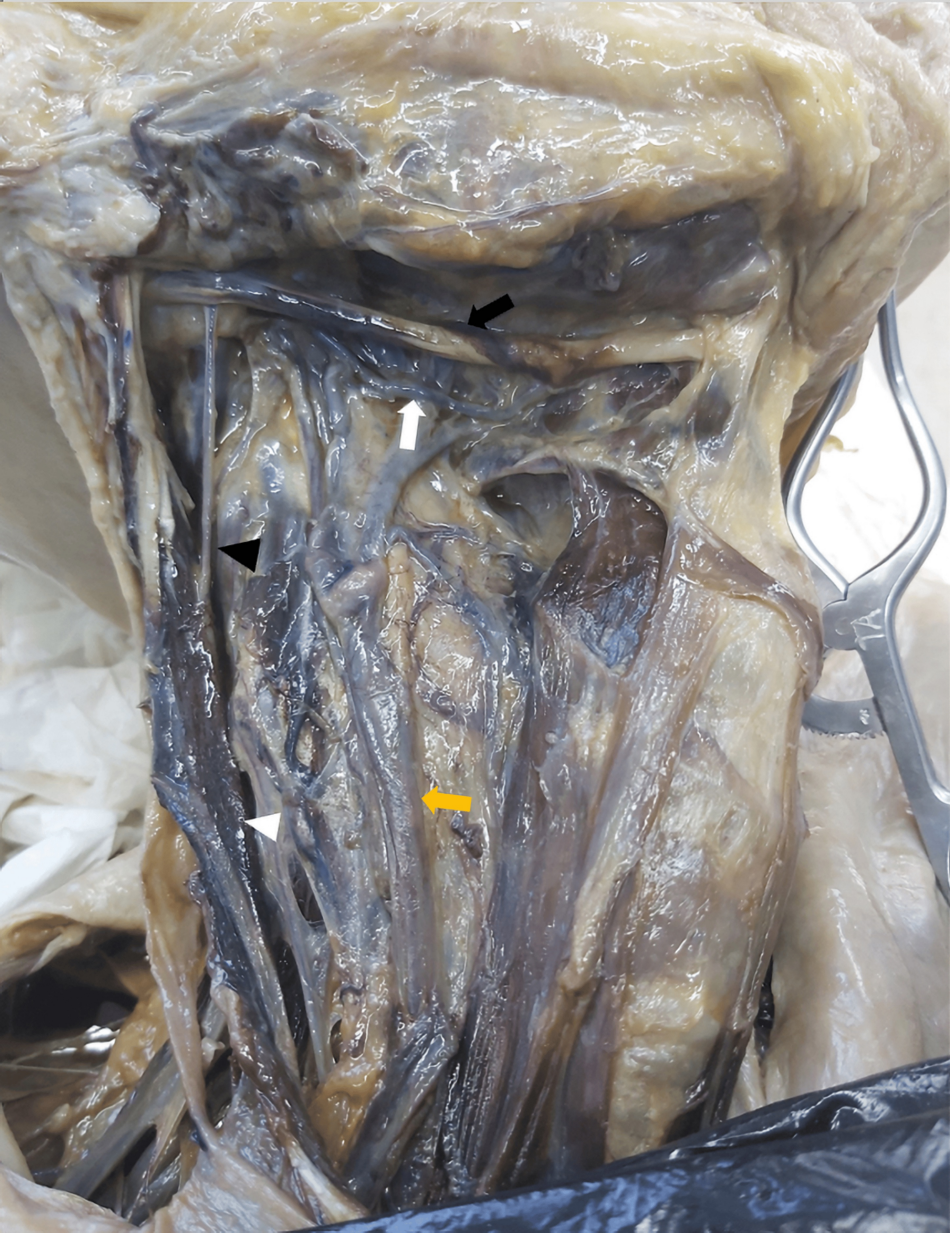
Hypoglossal nerve dissection. white arrow: hypoglossal nerve; black arrow: digastric muscle; orange arrow: internal jugular vein; black arrow head: accessory nerve; white arrow head: sternocleidomastoid muscle

The dissection of lower cranial nerves is shown in Figures 1, 2, 3, and their position in relation to important landmarks in the neck and distances from lower cranial nerves to important landmarks in the neck are described in Tables 1, 2, respectively.

**Table 1:** The position of lower cranial nerves in relation to important landmarks in the neck. The data has been presented as N (%). CCA: common carotid artery; IJV: internal jugular vein

		N (%)
Vagus nerve		
Relation with CCA		
	Posterolateral	10 (55.6)
	Lateral	6 (33.3)
	Posterior	2 (11.1)
Relation with IJV		
	Medial	10 (55.6)
	Posterior	8 (44.4)
Accessory nerve		
Relation with the IJV at the level of the jugular foramen		
	Anteromedial	8 (44.4)
	Posterior	5 (27.8)
	Medial	3 (16.7)
	Anterior	2 (11.1)
Relation with the IJV at the level of the posterior belly of the digastric		
	Posterior	9 (50.0)
	Lateral	4 (22.2)
	Anterior	3 (16.7)
	Anterolateral	1 (5.6)
	Posterolateral	1 (5.6)
Relation with IJV inferiorly to the digastric muscle		
	Posterolateral	14 (77.8)
	Anterolateral	4 (22.2)
Hypoglossal nerve		
Relation with the digastric muscle tendon	Inferior	9 (50.0)
	Medial	6 (33.3)
	Superior	3 (16.7)

**Table 2:** Distance from lower cranial nerves to important landmarks in the neck (mm). The data has been represented as Mean ± Standard Deviation or Median (minimum-maximum). The shortest distance between the nerves and each structure was measured in milimeters. IJV: internal jugular vein; SCM: sternocleidomastoid muscle

	Mean ± Standard Deviation	Median (min-max)
Vagus nerve		
Posterior belly of the digastric	6.94 ± 3.22	6.50 (2-15)
Lateral border of the hyoid bone	18.17 ± 7.00	17.50 (8-34)
Lateral border of the cricoid cartilage	15.78 ± 5.50	15.00 (8-27)
Accessory nerve		
Length from the jugular foramen until crossing the IJV	17.50 ± 7.80	20.50 (6-30)
Location where it perforates the SCM	49,83 ± 11,43	45.00 (38-80)
Hypoglossal nerve		
Carotid bifurcation	23.94 ± 9.90	27.00 (11-45)
Lateral border of the hyoid bone	10.24 ± 5.03	10.00 (3-17)
Lateral border of the cricoid cartilage	67.06 ± 11.90	69.50 (43-81)
Facial nerve bifurcation	18.76 ± 3.42	19.00 (12-26)
Lingual artery origin	7.41 ± 3.04	8.00 (2-13)
Occipital artery origin	9.44 ± 7.42	7.00 (1-29)
Ansa cervicalis origin		
Carotid bifurcation	27.64 ± 8.50	27 (15-51)
Angle of the mandible	27.50 ± 11.06	25 (10-55)

Vagus nerve

The CN X was posterolateral to the CCA in 10 (55.6%), lateral in six (33.3%), and posterior in two (11.1%) dissections. Its relative position to the IJV was medial in 10 (55.6%) and posterior in eight (44.4%) cases.

During its course, the mean distance of the CN X to the posterior belly of the digastric was 6.94±3.22 mm, to the hyoid bone was 18.17±7 mm, and to the lateral border of the cricoid cartilage was 15.78±5 mm.

Accessory nerve

At the jugular foramen, the CN XI was anteromedial in eight (44.4%), posterior in five (27.8%), medial in three (16.7%), and anterior in two (11.1%) cases in relation to the IJV. At the level of the posterior belly of the digastric, it was located posterior in nine (50%), lateral in four (22.2%), anterior in three (16.7%), anterolateral in one (5.6%), and posterolateral in one (5.6%) to the IJV. Below the digastric muscle, the CN XI was posterolateral to the IJV in 14 (77.8%) and anterolateral in four (22.2%) cases.

The mean length of the CN XI from the jugular foramen until it crosses the IJV was 17.50±7.8 mm. Furthermore, the CN XI has perforated the SCM 49.83±11.43 mm distal to the muscle’s proximal fixation.

Hypoglossal nerve

The CN XII coursed inferiorly to the digastric muscle tendon in nine (50%), medial in six (33.3%), and superior in three (16.7%) cases.

During its course, the average distance of the CN XII to the carotid bifurcation was 23.94±9.90 mm, to the hyoid bone was 10.24±5.03 mm, to the lower border of the cricoid cartilage was 67.06±11.90 mm, to the CN XII bifurcation was 18.76±3.42 mm, to the origin of the lingual artery, was 7.41±3.04 mm, and to the origin of the occipital artery was 9.44±7.42 mm.

The origin of the ansa cervicalis is distanced on average 27.64±8.50 mm from the carotid bifurcation and 27.50±11.06 mm from the angle of the mandible.

Laterality

There were no differences in any of the previously determined distances or anatomical relations between lower cranial nerves and important neck landmarks when the right and left sides were compared.

Gender

A comparison of female and male measurements between lower cranial nerves and important neck landmarks showed that the distance from the origin of the occipital artery to the CN XII was superior in female cadavers with a median of 7 (6-29) mm when compared to 4 (1-13) mm in male dissections (p = 0.012; Mann-Whitney U test). Furthermore, the distance from the carotid bifurcation to the CN XII was superior in male cadavers, with a mean of 31.71±6.78 mm in comparison to 18.50±7.92 mm in female necks (p<0.001; student’s T test). There were no other differences in measurements or anatomical relationships found between genders.

Age

Older age was associated with a higher distance from the CN XII to the lower border of the cricoid cartilage (p = 0.015; Pearson’s r = 0.595; Pearson correlation coefficient test). There were no other associations between age and anatomical relationships or measurements involving lower cranial nerves.

## Discussion

Thiel-embalmed cadavers were used to analyze distances and anatomical relations between important neck landmarks and the lower cranial nerves. When compared to fresh frozen cadavers, the Thiel embalming method allows the cadavers to be used on multiple occasions and for longer periods of time [13]. Furthermore, Thiel embalming leads to more realistic tissue conservation, retaining the natural color and consistency of the tissues [13,14], and was proven to be a more realistic neck surgery training model [15] in comparison with the traditional formaldehyde fixation.

In this study, the CN X was more commonly posterolateral to the CCA and medial to the IJV, with variations in up to 45% of the cases. It was previously reported to be mainly lateral to the CCA (94.3%) in fresh frozen cadavers [11] and to be more frequently posterior to the CCA (60%) in formaldehyde-embalmed cadavers [16]. These differences might be explained by the utilization of different conservation methods. The mean distances of the CN X to the posterior belly of the digastric (6.94 mm), the hyoid bone (18.17 mm), or the lateral border of the cricoid cartilage (15.78 mm) were similar to what was previously reported (8.4±3 mm, 20.8±7.4 mm, and 23.7±11.2 mm, respectively) [11].

In relation to the IJV, the CN XI was more commonly anteromedial at the jugular foramen, posterior at the posterior belly of the digastric, and posterolateral below this muscle, with a significant rate of anatomical variations. As in most anatomical studies, a previous review categorized the relationship of the CN XI to the IJV as lateral (including lateral, anterior, or superficial positions), medial (including medial, posterior, or deep positions), traversing, or dividing around the vein. This review included 1070 in vivo or cadaveric neck dissections and has shown that the CN XI runs lateral to the IJV vein in most studies, with a prevalence that ranged from 39.8% to 96.6% [17]. Furthermore, in a study with 207 patients submitted to neck dissection, the CN XI was found to be more commonly lateral/superficial (95.6%) [18], while cadaveric studies showed a more prevalent medial/deep position of the nerve [19,20]. Taylor et al. hypothesized that in cadaveric studies the IJV is commonly partially collapsed and that anatomists frequently dissect the nerve, tracing it to the skull base, where it is frequently medial to the IJV, which could justify a more prevalent medial position [18]. Thus, it is important to precisely clarify in every study at which level the nerve was identified and which relation categories are being used in order to better comprehend the anatomical relationships of the CN XI.

The relationship of the CN XI with the IJV at the posterior belly of the digastric is important since it is frequently found at this level in vivo neck dissections [18]. In a study with 61 formalin-embalmed cadavers, the CN XI was anterior to the IJV in most cases (80%) [19], and in another study with 39 fresh-frozen cadavers, it was found to be more commonly lateral (55.7%) to the IJV at the same level [11]. In our study, the CN XI was more frequently posterior to the IJV at this level, which could possibly be explained by a smaller sample size, racial/ethnic differences, or the use of Thiel-embalmed cadavers in contrast to fresh-frozen or formalin-embalmed cadavers.

The mean length of the CN XI from the jugular foramen until it crosses the IJV was 17.50±7.8 mm, which is slightly inferior to what was previously described in a cadaveric dissection (2.38 cm) [19] or in vivo (2.34 cm) studies [21]. Moreover, the CN XI has perforated the SCM 49.83±11.43 mm distal to the muscle’s mastoid origin, and during this length, it could be susceptible to injury while dissecting medially to the SCM. To our knowledge, no other study has measured this distance.

The CN XII has traveled mainly inferiorly to the digastric muscle tendon and was, less frequently, medial or superior to the latter. In an anatomical study with formaldehyde-embalmed cadavers, the CN XII was also mainly inferior to the digastric tendon in 54.3% of the cases [22]. The distance from the carotid bifurcation to the CN XII varies across studies, with results going from 14.69 mm to 27.7 mm [11,16,22], including 23.94±9.90 mm registered in our dissections. Furthermore, the CN XII was shown to be significantly closer to the CCA bifurcation when the CCA bifurcates at the level of the hyoid bone compared to when it bifurcates at the superior border of the thyroid cartilage [16]. As such, variations in the CN XII-CCA bifurcation distance seem to be more closely associated with different carotid bifurcation levels than with the nerve position itself. In addition to this, the CN XII courses close to the ICA and ECA, where it travels less than 1 cm away from the origin of the lingual and occipital arteries, leading to its vulnerability to injury in surgeries to the carotid arteries [5].

The distance from the CN XII to the CN VII bifurcation is important to plan CN XII to CN VII anastomosis. In this study, the CN XII had a mean distance of 18.76 mm to the CN VII bifurcation, similar to 17.3±2.5 mm reported by Asaoka et al. [23] and 16.1±3.3 mm reported by Salame et al. [22]. Furthermore, Salame et al. found previously that the length of the CN VII from the stylomastoid foramen to the bifurcation was 16.44 mm, suggesting that, in the majority of the cases, a tension-free neurorrhaphy is possible with mobilization of the CN VII and the CN XII without the need of a mastoidectomy [24].

The ansa cervicalis to recurrent laryngeal nerve anastomosis is gaining popularity for the treatment of unilateral recurrent laryngeal nerve paralysis in selected patients [25]. For the success of this surgery, it is important to understand the anatomy of the ansa cervicalis and its variations to find it successfully without injuries to the nerve or nearby structures. However, the anatomy of this nerve is highly inconsistent, with many variations in the level of origin of the superior root from CN XII; it can travel inside or outside the carotid sheath, and it may have different loop levels, among other variations [26,27]. We have found the ansa cervicalis to originate 27.64±8.50 mm from the CCA bifurcation and 27.50±11.06 mm from the angle of the mandible, with its respective standard deviations showing a high variability in the origin of this nerve.

As Yigit et al [11], we have found no differences when comparing right and left lower CN's neck relations and distances to neck landmarks. This shows that both sides of the neck often have similar anatomy, which may be helpful when performing bilateral neck surgeries such as bilateral neck dissections. Furthermore, we have compared lower cranial nerve relations to anatomical landmarks between males and females, and we have found a higher distance from the origin of the occipital artery to the CN XII in female cadavers and a higher distance from the carotid bifurcation to the CN XII in male necks. We hypothesize that the CCA bifurcation may lie inferiorly in males when compared to females, which may explain both a higher CCA bifurcation-CN XII distance in males and a higher occipital artery origin-CN XII distance in females, where the CN XII courses more often inferiorly to the occipital artery origin. Lastly, the distance between the lower cricoid border and the CN XII increases with age, which suggests that laryngeal structures may continue to descend with age even after adulthood.

This study has some limitations. Firstly, there was a small sample size due to the availability of Thiel-embalmed cadavers in our department. A larger sample would be needed to confirm our results. Lastly, even though measurements were rigorous and confirmed by both authors, measurements were performed with the MedBAR® Skin Marker Scale, which is accurate up to 1 mm. There are other measurement techniques, such as using a measuring probe and reading against a digital caliper, which could be more accurate. On the other hand, we believe that using the MedBAR® Skin Marker Scale did not affect our results.

## Conclusions

This study shows that there is a high variability in the course of the lower cranial nerves in the neck and their relations to important anatomical structures, and some of these variations might be related to age or gender.

Every surgeon performing neck procedures should be familiar with these anatomical relations and variations in order to avoid iatrogenic injury. Anatomical studies may vary in many aspects, which can also lead to different conclusions.
